# DNA Compaction and Charge Inversion Induced by Organic Monovalent Ions

**DOI:** 10.3390/polym9040128

**Published:** 2017-03-30

**Authors:** Wenyan Xia, Yanwei Wang, Anthony Yang, Guangcan Yang

**Affiliations:** School of Physics and Electronic Information, Wenzhou University, Wenzhou 325035, China; xiawenyan147@163.com (W.X.); wangyw@wzu.edu.cn (Y.W.); yanggc77@gmail.com (A.Y.)

**Keywords:** DNA compaction, charge inversion, monovalent ions, hydrophobic effect

## Abstract

DNA condensation and charge inversion usually occur in solutions of multivalent counterions. In the present study, we show that the organic monovalent ions of tetraphenyl chloride arsenic (Ph_4_As^+^) can induce DNA compaction and even invert its electrophoretic mobility by single molecular methods. The morphology of condensed DNA was directly observed by atomic force microscopy (AFM) in the presence of a low concentration of Ph_4_As^+^ in DNA solution. The magnetic tweezers (MT) measurements showed that DNA compaction happens at very low Ph_4_As^+^ concentration (≤1 μM), and the typical step-like structures could be found in the extension-time curves of tethering DNA. However, when the concentration of Ph_4_As^+^ increased to 1 mM, the steps disappeared in the pulling curves and globular structures could be found in the corresponding AFM images. Electrophoretic mobility measurement showed that charge inversion of DNA induced by the monovalent ions happened at 1.6 mM Ph_4_As^+^, which is consistent with the prediction based on the strong hydrophobicity of Ph_4_As^+^. We infer that the hydrophobic effect is the main driving force of DNA charge inversion and compaction by the organic monovalent ion.

## 1. Introduction

DNA is one of the most important biological polyelectrolytes, and is highly negatively charged in solution. The highly-charged stiff polymer can be condensed into compact structures by multivalent ions and many other condensing agents [1,2,3]. The understanding of DNA compaction is not only important for the study of fundamental biological processes such as chromosome compacting, but also for the development of new gene carriers in therapeutic applications [4,5,6]. On the other hand, DNA compaction is closely related with its charge screening, because structural packaging requires an effective screening of the negative charges on DNA. The process is generally considered to be related to the neutralization—or more likely overcompensation—of the DNA electric charge [7,8,9]. Overcompensation or charge inversion occurs when the charge of counterions surrounding the DNA surface are greater than the bare charge of polyelectrolyte itself. For DNA systems, the effect seems to be found almost exclusively in the case of multivalent ions in aqueous solution [7,10,11,12,13,14]. Charge inversion was also observed in mixtures of charged polyelectrolytes with oppositely charged oligomers [7,15], polymers [16,17,18,19,20,21,22,23,24], colloids [18,25,26,27,28], or micelles [29]. The related DNA compaction from bulk solution critically depends on the valence of the counterions, and a valence of three or larger is required to overcome the inherently large electrostatic repulsive barrier between the like-charged polyelectrolytes [30,31,32,33].

However, the underlying microscopic mechanism of attraction between like-charged macroions such as DNA and their charge inversion is still controversial. There have been a number of theoretical studies aimed at elucidating the fundamental physical mechanisms responsible for DNA compaction and charge inversion [34,35]. All these systems are strongly correlated; the electrostatic interactions are strong and the effects of thermal motions, translational, or conformational entropy of chains is small. It has been proposed that electrostatic correlations are dominant and lead to charge inversion in the case of counterions with valences larger than two near strongly-charged interfaces [8]. In the case of monovalent ions, electrostatic correlations are weak and no charge inversion is expected. Recently, Martin-Molina et al. [36] proposed a new mechanism for the charge inversion of colloids in electrolyte solutions based on the hydrophobic effect. They observed charge inversion due to the organic monovalent ion Ph_4_As^+^ in colloids, and the effect was attributed to the hydrophobic effect. In the present study, we introduce the organic monovalent ion Ph_4_As^+^ into a DNA system and find that it not only provokes the charge inversion of DNA, but also leads to DNA compaction, which is the first experimental evidence for DNA compaction induced by monovalent cations. The phenomenon seems to be similar to the poor solvent effect of neutral condensing agents such as ethanol in DNA solution [37].

## 2. Experimental Procedures

### 2.1. Materials

Tetraphenylarsonium chloride (Ph_4_AsCl) is a tetrahedron consisting of a central As atom covalently bonded to four hydrophobic groups (the phenyl –C_6_H_5_ rings). This highly hydrophobic cation is widely employed as a reference cation in electrochemistry because of the relatively small importance of electrostatics in its hydration free energy in different media; this is due to its large size (diameter *d* = 0.94 nm), its symmetry, and its monovalent character. Tetraphenylarsonium chloride (C_24_H_20_AsCl·HCl·xH_2_O), sodium bromide (NaBr), and MgCl_2_ were purchased from Sigma-Aldrich, (Sain Louis, MO, US). Bacterial *λ*-DNA was purchased from New England Biolabs (Ipswich, MA, US), and it had a concentration of 500 ng/μL as obtained from the manufacturer. We could use *λ*-DNA directly without further treatment in atomic force microscopy (AFM), but DNA modification was necessary before pulling in magnetic tweezers (MT). The solvent we used was 1 mM sodium bromide aqueous solution, and water was deionized and purified by a Millipore system and had a conductivity less than 1 × 10^–6^ Ω^–1^ cm^–1^. Mica for AFM imaging was cut into approximately 1 cm^2^ square pieces, and their surfaces were always freshly cleaved before use. All chemical agents were used as received, and all measurements were repeated at least twice to obtain consistent results.

### 2.2. AFM Imaging

The sample preparation procedure is similar to the one in our previous work [36]. It can be briefly described as follows: mica disks of diameter one centimeter attached to magnetic steel disks were used as substrates for DNA adsorption. For each sample, the final concentration of DNA was 1 ng/μL, corresponding to 3 μM of phosphate groups, and a drop of about 15 μL of Ph_4_As^+^ mixture was deposited for 3 min on a freshly-cleaved mica surface. The surface was rinsed with distilled water and dried with a gentle flow of nitrogen gas.

A multi-mode atomic force microscope (SPM-9600, Shimadzu, Kyoto, Japan) was used for DNA imaging in the presence of Ph_4_As^+^. All AFM images were captured in the conventional ambient tapping mode, with scan speeds of ≈2 Hz and data collection at 512 × 512 pixels. All the images were processed manually using off-line analysis software equipped with the microscope.

### 2.3. Magnetic Tweezers Experiment

**Figure 1:**
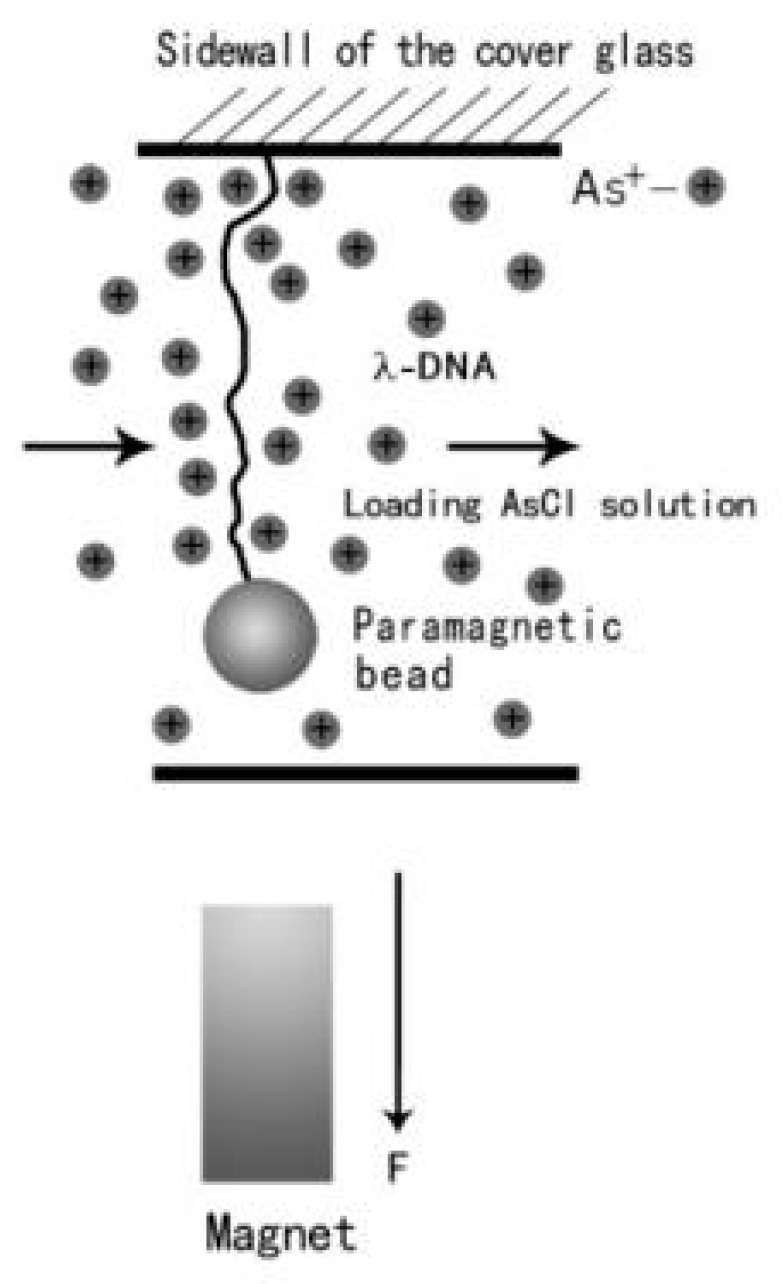
A schematic diagram of magnetic tweezers.

Magnetic tweezers are common tools for manipulating single molecules, such as tethering a condensed DNA molecule [38]. A transverse MT system was composed of an inverted microscope and charge coupled device (CCD) controlled by a personal computer, which is schematically shown in Figure 1. In our experiments, the ends of DNA were coated with biotin and digoxin by biochemical method, and the surfaces of paramagnetic beads were covered with streptavidin. A coverslip with one side polished was sandwiched between two glass slides, and was used as a flow chamber by sealing the sealing the open sides of the structure. The polished sidewall was silylated and functionalized with anti-digoxin in order to link with the dig-end of DNA. The other end of DNA was linked to a paramagnetic bead by the streptavidin–biotin bond. The force was applied to the paramagnetic bead by adjusting the permanent magnet which is installed on a micromanipulator. A video camera was used to monitor the image of the tethered structure, and the positions of paramagnetic beads were recorded in real-time. The analysis of the extension and force was determined by a tracking algorithm based on correlation function [39].

Various concentrations of Ph_4_AsCl solution were mixed with NaBr solution (1 mM), then an equal volume of DNA solutions containing 10 mM MgCl_2_ were added for magnetic tweezers measurement. The solution was incubated for 30 min at least at room temperature, and introduced into the flow cell by using a syringe pump. In a typical measurement, we moved the magnet from some distance to some position close to a paramagnetic bead, thus applying a magnetic force on the suspended bead. When a fixed magnetic force was applied to the bead, we monitored the end-to-end length of DNA in real-time to measure its conformational change.

### 2.4. Electrophoretic Mobility Measurement by Dynamic Light Scattering (DLS)

The electrophoresis-mobility measurements were carried out by using a dynamic light scattering device of Malvern Zetasizer Nano ZS90 (Malvern Instruments Ltd., Worcestershire, UK) equipped with the patented M3-PALS technique. The laser source is a He-Ne gas laser (*λ* = 633 nm) and the light scattering is collected by an avalanche photodiode mounted on the goniometer arm to the direction of the incident radiation. The DNA samples were diluted to a concentration of 1 ng/μL in a buffer solution containing 1 mM NaBr and 5 mM MgCl_2_; then, different concentrations of Ph_4_As^+^ were added. All measurements were carried out after 5 min incubation at room temperature. During the measurement, 1 mL of DNA solution was used, and the sample cell was kept at 25 °C temperature.

## 3. Results and Discussion

### 3.1. Observation by AFM

**Figure 2:**
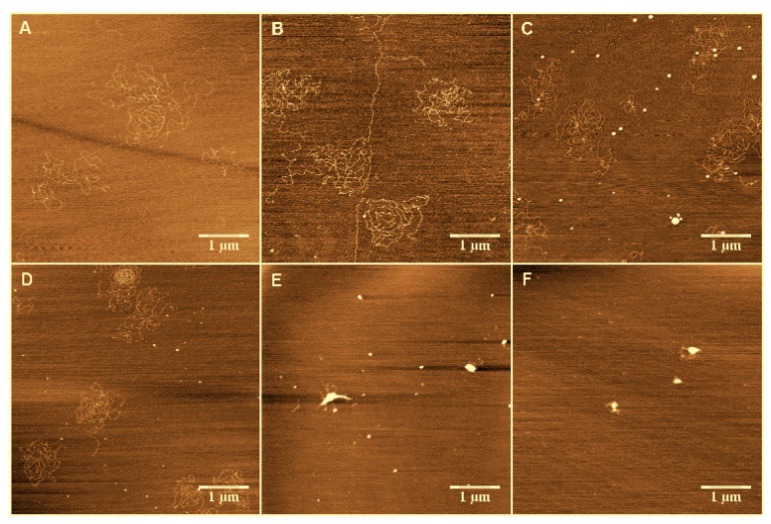
AFM observation of DNA complexes. Panel (**A**) λ-DNA in 5 mM Mg^2+^ solution; Panels (**B**–**F**) DNA conformations at different concentrations (0.01, 0.05, 0.1, 0.5 and 1 mM, respectively) of As^+^ in 5 mM Mg^2+^ buffer.

We attempted to observe the change in morphology of the DNA–As^+^ complexes in the presence of different concentrations of Ph_4_As^+^ in solution. In our experiment, MgCl_2_ solution (5 mM) was added into the buffer to adsorb DNA on the mica surface. The AFM images of DNA–As^+^ complexes on the mica surface are shown in Figure 2. For reference, we start by imaging DNA alone in the same buffer condition as used for the complexes (Figure 2A). We can see that the DNA molecules are well separated on the surface, and have relaxed morphologies with no compaction loops. From Figure 2B–F, the corresponding molar ratio between arsenic cations and the phosphate group of DNA varies from 3.33 to 333.3. As shown in Figure 2B, there are few intermolecular contacts at lower As^+^ concentrations, but individual molecules have an increased number of intramolecular loops with increasing As^+^ concentration. However, when [As^+^] > 0.1 mM, typical condensed structures were observed, and a part of the DNA structures were still in the coiled conformation. A condensed center appeared (Figure 2C), and DNA highly looped around this point, as shown in Figure 2D. The compaction grew gradually when the concentration of As^+^ increased further (Figure 2E), and at the highest concentration (Figure 2F), we can see even more compacting patterns (e.g., globules) on the mica surface.

AFM may modify the actual morphology of DNA in As^+^ solution. We have previously shown that the treatment of condensed DNA adsorbed to a surface only reduces the molecules’ heights but maintains their lateral dimensions [37]. Thus, AFM images flatten the morphology of DNA condensates with their original compacting structure. However, the condensed DNA is adsorbed on a mica surface and only binds to the surface loosely. Despite the interactions between the substrate and the polyelectrolytes (which can modify the structure of complexes), there is a nice correlation between the macroscopic phase diagram and the AFM observations. We think that the reason for this phenomenon is similar to the mechanism of alcohol [37] leading to DNA compaction. Since DNA is a semiflexible polymer molecule and the attractive forces between DNA segments are rather weak, the DNA molecules in poor solvent tend to form compact toroidal structures in solution due to the equilibrium between the exclusion and bending energy.

**Figure 3:**
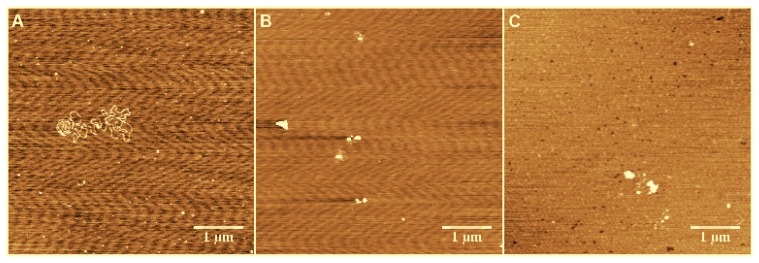
The structure of DNA in mixture solution of various concentrations ((**A**) 0.1 mM; (**B**) 0.5 mM; (**C**) 1 mM) of As^+^ and 1 mM Mg^2+^.

**Figure 4:**
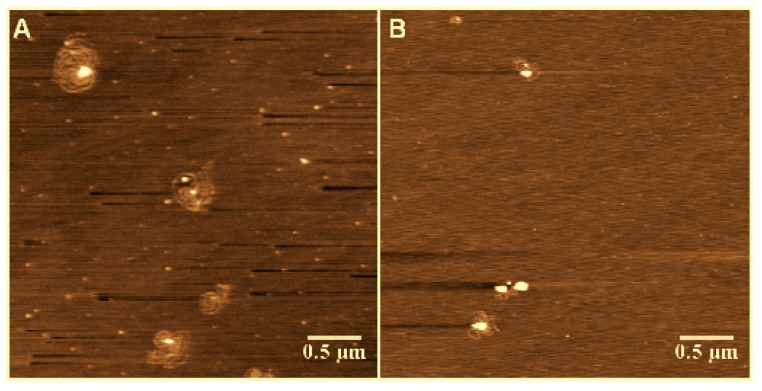
DNA structures in solution of (**A**) 0.01 mM spermine and (**B**) 0.5 mM As^+^.

To enhance DNA adsorption on the mica surface for imaging, we used a buffer containing a high concentration of Mg^2+^ (5 mM). To rule out its effect on DNA compaction, we performed the same experiments in 1 mM of Mg^2+^—the lowest concentration to deposit DNA on a mica surface. The results are similar, and are shown in Figure 3. We can see that the DNA condensed from loose structures to highly-compact globules with increasing As^+^ concentration in the low concentration of Mg^2+^. Thus, we can deduce that the DNA compaction is caused by arsenic ion rather than the effect of magnesium ion. Actually, the DNA condensing ability of Ph_4_As^+^ is much weaker than spermine, as shown in Figure 4, where the effect of 0.5 mM As^+^ is comparable with 0.01 mM spermine.

### 3.2. Tethering of Single DNA Molecules

**Figure 5:**
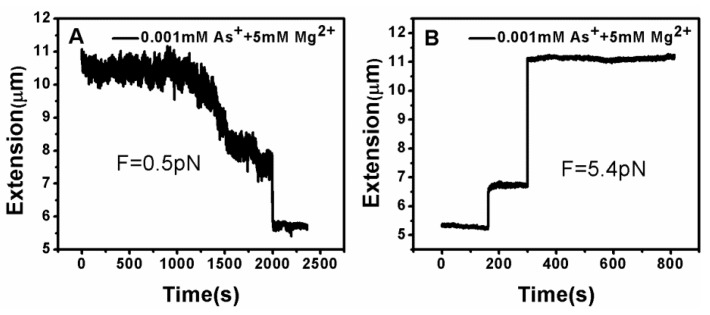
DNA extension–time curves under the influence of Ph_4_As^+^. (**A**) The shrinking of DNA at pulling force 0.5 pN; (**B**) unraveling of DNA condensed structures.

**Figure 6:**
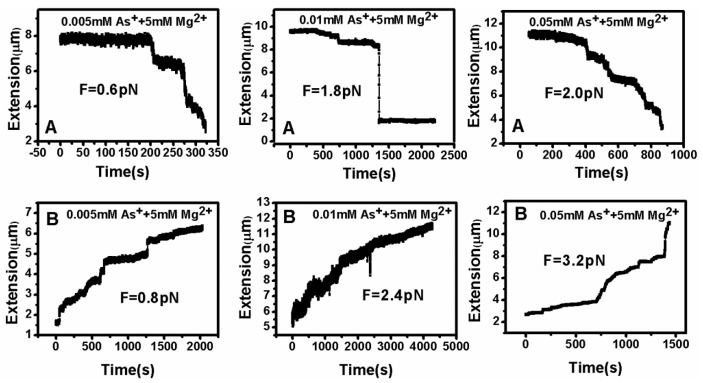
DNA extension–time curves when (**A**) contracting and (**B**) stretching at various Ph_4_As^+^ concentrations.

**Figure 7:**
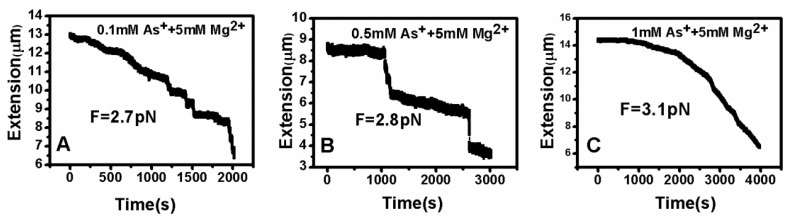
DNA extension–time curves at high concentrations ((**A**) 0.1 mM; (**B**) 0.5 mM; (**C**) 1 mM) of Ph_4_As^+^.

DNA tethering was achieved in a magnetic tweezers (MT) setup, as described above. We first put the microsphere-bound DNA molecules in NaBr buffer (1 mM) and magnesium ion (5 mM) into the flow cell and incubated them for 30 min at room temperature. Then, we could find that some paramagnetic beads had tethered to the surface of the sidewall through a single DNA molecule. In the absence of any condensing agents, we found that the extension of DNA was close to 16 μm under high extension (>10 pN). Then, about 400 μL of different concentrations of Ph_4_AsCl solution were loaded into the flow cell and stretched the DNA by approaching the magnet to the bead, or releasing the bead by moving back the magnet while the extension of the DNA molecule was monitored in real-time. The results of the DNA tethering are shown in Figure 5 and Figure 6. From Figure 5A, we can see the stepwise shrinking of the DNA extension when a constant 0.5 pN pulling force was exerted upon the bead at low concentration of Ph_4_AsCl (1 μM). After the DNA molecule condensed to a compact state, we had to apply much larger force (5.4 pN in this case) to unravel the condensed structure, as shown in Figure 5B. We could repeat the shrinking and unraveling many times, and the processes was reversible in our experiment. When the concentration of Ph_4_AsCl in the buffer increased, the tethering force increased accordingly to unravel the condensed DNA structures. We could still see the stepwise structure in the shrinking or pulling curves of DNA molecules when the concentration of Ph_4_AsCl is not greater than 0.5 mM, as shown in Figure 6 and Figure 7. However, the stepwise structure disappeared in the shrinking curves when the concentration of As^+^ reached 1 mM, as shown in Figure 7C. We infer than a more compact structure (e.g., globule) was formed in the high concentration condition.

**Figure 8:**
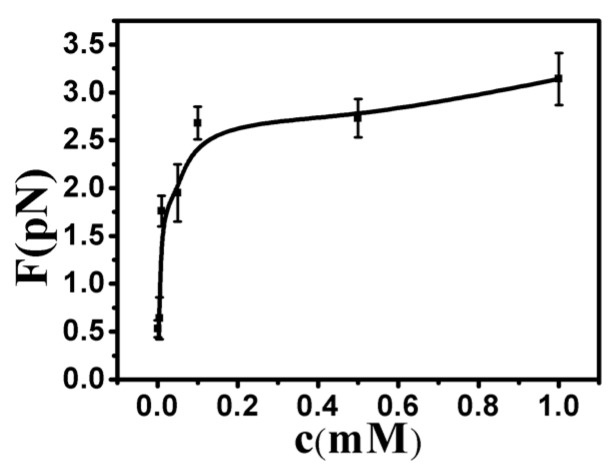
The curve of condensing force at different concentrations of Ph_4_As^+^.

By analyzing the tethering curves, we concluded that the hydrophobic driving force mechanism is similar to the effect of poor solvent. However, the former is much stronger, since the tethering force is on the order of a few pN, while the shrinking force is difficult to measure in the case of ethanol solution [35]. For consistency and reproducibility, we repeated our tethering cycle under each condition at least 10 times. The curve of condensing force is presented in Figure 8. The details of pulling forces and DNA particle sizes are presented in Tables S1–S3.

### 3.3. Electrophoretic Mobility of DNA

**Figure 9:**
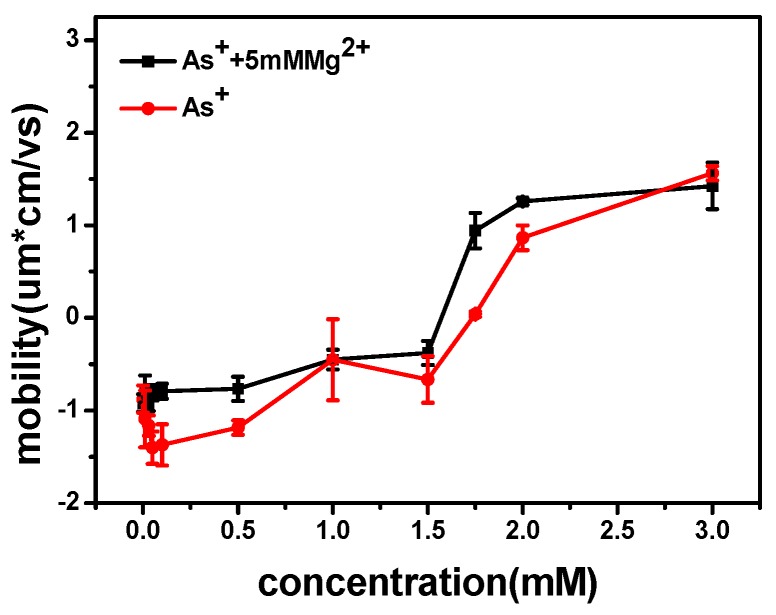
Electrophoretic mobility of DNA vs. the concentration of Ph_4_As^+^.

We measured the electrophoretic mobility of DNA in the mixture solution with different concentrations of Ph_4_As^+^ and 5 mM MgCl_2_ by dynamic light scattering technique. The result is shown in Figure 9. We can see that the mobility of DNA changed from negative to positive values with the increasing As^+^ concentration. The mobility approached zero at about 1.75 mM of Ph_4_As^+^. For cation concentrations larger than this, the mobility reversed from negative to positive, implying that charge inversion occurred. Meanwhile, the size of DNA complex decreased with As^+^ concentration. When the concentrations of As^+^ were 0.01, 0.05, 0.1, 0.5, 1, 2, and 3 mM, the corresponding particle sizes of DNA condensates were 310, 300, 210, 200, 180, 182, and 180 nm, respectively, and the errors were ±20 nm.

The mechanism of DNA charge reversal is similar to colloid charge inversion, the hydrophobic effect as a driving force for charge inversion. This mechanism is able to induce charge inversion at low concentrations of monovalent ions containing hydrophobic functional groups, and saturation effects appearing at higher concentrations.

Following the similar analysis in Reference [36], the critical concentration of counterions in solution when charge inversion occurs can be expressed as

(1) $$c_{B}^{I}=\frac{\left|\sigma_{0}\right|}{ed}\exp(\Delta \mu^{0}/k_{B}T)$$

where $\Delta \mu^{0}\;=\;\mu_{S}^{0}-\;\mu_{B}^{0}$ is the difference in free energy between the dehydrated ion at close proximity to the colloid and the hydrate ion in bulk electrolyte, *d* is the diameter of the counter ions, and *σ*_0_ is the bare charge density of DNA. For the current system, where *d* ≈ 1 nm, hydration energy is about –5*k*_B_*T*, and bare surface charge is about 1.0 e/nm^2^, one obtains $c_{B}^{I}=1.6\;\mathrm{mM}$. The measured value of charge inversion is about 1.75 mM, as shown in Figure 9, where charge inversion is observed for DNA in water at 298 K in the presence of low concentrations of a monovalent large organic cation [36]. The observed critical concentration is in agreement with the predicted value by hydrophobicity. It is noticeable that Equation (1) might only be applicable for a weakly charged system. DNA is a highly charged system where the correlation effect is significant and a more sophisticated theory is needed. In spite of the restriction, we can still use it as a preliminary estimation.

## 4. Conclusions

In summary, we found for the first time that the organic monovalent ions of tetraphenyl chloride arsenic (Ph_4_As^+^) can induce DNA compaction and its charge inversion, which is contradictory to the common understanding that charge inversion occurs only if the valence of counterions is *Z* ≥ 3. These findings were confirmed by magnetic tweezers measurements, atomic force microscopy (AFM) imaging, and dynamic light scattering (DLS).

DNA compaction is usually involved the first-order phase transition between elongated and compact states, although some chemical species cause shrinkage of DNA to be much different from the transition [40]. DNA compaction occurs in one mode or mixed modes among all-or-none compaction, progressive compaction, and adsorption and wrapping, depending on condensing agents and solvents. The DNA compaction in Ph_4_As^+^ solution is in a progressive compaction mode, which is directly shown in their AFM images (Figure 2 and Figure 3). Since the electrostatic interaction between monovalent ions and DNA is not able to induce its compaction or charge inversion, we infer that the main driving force of DNA charge inversion and compaction by the organic monovalent ion Ph_4_As^+^ is the hydrophobic effect.

## Supplementary Materials

The following are available online at www.mdpi.com/2073-4360/9/4/128/s1, Table S1: The condensing force of DNA at different concentrations of Ph_4_As^+^, Table S2: The unravelling force of DNA at different concentrations of Ph_4_As^+^, Table S3: The particle size of DNA at different concentrations of Ph_4_As^+^.
