# Supplementary material for: DNA Compaction and Charge Inversion Induced by Organic Monovalent Ions
Source: Polymers (Basel). 2017 Mar 30;9(4):128. doi: 10.3390/polym9040128 (PMC6431901; doi:10.3390/polym9040128)
Supplement: Supplementary file 1 [file polymers-09-00128-s001.pdf]

Supplementary Materials: DNA Compaction and Charge Inversion Induced by Organic Monovalent Ions

Wenyan Xia, Yanwei Wang, Anthony Yang and Guangcan Yang

Tables S1 and S2 show the condensing force and unravelling forces of DNA at various concentrations of Ph_4As^+, which were measured by tethering DNA in flow cells of magnetic tweezers. The average values of both condensing and unravelling forces increased with the concentration of arsenic ions, indicating the tendency of DNA compaction. The particle sizes of DNA in Table S3 show the similar compacting trend.

Table S1. The condensing force of DNA at different concentrations of Ph_4As^+.

Concentration (mM)	Unravelling force (pN)										Average value
0.005	0.90	0.87	0.81	0.79	0.84	0.77	0.86	0.82	0.79	0.85	0.83
0.01	2.36	2.38	2.41	2.40	2.33	2.34	2.42	2.31	2.39	2.46	2.38
0.05	3.21	3.24	3.13	3.20	3.19	3.11	3.21	3.14	3.23	3.24	3.19

Table S2. The unravelling force of DNA at different concentrations of $Ph4As^+$.

Concentration (mM)	Condensing force (pN)										Average value
0.001	0.51	0.48	0.60	0.62	0.43	0.54	0.51	0.46	0.53	0.62	0.53
0.005	0.58	0.72	0.61	0.59	0.71	0.65	0.56	0.67	0.62	0.69	0.64
0.01	1.68	1.73	1.82	1.78	1.74	1.84	1.73	1.69	1.78	1.81	1.76
0.05	1.88	2.03	2.02	1.89	2.10	2.01	1.99	1.86	1.87	1.85	1.95
0.1	2.73	2.65	2.69	2.71	2.68	2.70	2.74	2.61	2.67	2.62	2.68
0.5	2.83	2.80	2.76	2.61	2.69	2.81	2.79	2.74	2.63	2.64	2.73
1	3.07	3.12	3.18	3.21	3.16	3.11	3.24	3.11	3.05	3.15	3.14

Table S3. The particle size of DNA at different concentrations of Ph_4As^+.

Concentration (mM)	Particle size (nm)					Average value
0.01	313	308	309	311	309	310
0.05	297	296	301	306	300	300
0.1	214	211	207	206	212	210
0.5	206	198	203	197	196	200
1	187	183	176	178	176	180
	186	185	178	173	188	182
3	176	183	180	178	183	180
